# Does dapagliflozin regress left ventricular hypertrophy in patients with type 2 diabetes? A prospective, double-blind, randomised, placebo-controlled study

**DOI:** 10.1186/s12872-017-0663-6

**Published:** 2017-08-23

**Authors:** Alexander J.M. Brown, Chim Lang, Rory McCrimmon, Allan Struthers

**Affiliations:** 1 Cardiovascular Medicine, Division of Molecular and Clinical Medicine, Medical Research Institute, Ninewells Hospital and Medical School, Mailbox 2, Dundee, DD1 9SY UK; 2 Cardiology, Division of Molecular and Clinical Medicine, Medical Research Institute, Ninewells Hospital and Medical School, Mailbox 2, Dundee, DD1 9SY UK; 3 Experimental Diabetes and Metabolism, Division of Molecular and Clinical Medicine, School of Medicine, Level 5, Ninewells Hospital and Medical School, Mailbox 12, Dundee, DD1 9SY UK; 4 Cardiovascular Medicine and Therapeutics, Division of Molecular and Clinical Medicine, Medical Research Institute, Ninewells Hospital and Medical School, Mailbox 2, Dundee, DD1 9SY UK

**Keywords:** Diabetes, SGLT2 inhibitor, Left ventricular hypertrophy, Mechanistic trial, Cardiac MRI

## Abstract

**Background:**

Patients with diabetes have a two to fourfold increased risk for development of and death from cardiovascular disease [CVD]. The current oral hypoglycaemic agents result in limited reduction in this cardiovascular risk. Sodium glucose linked co-transporter type 2 [SGLT2] inhibitors are a relatively new class of antidiabetic agent that have been shown to have potential cardiovascular benefits. In support of this, the EMPA-REG trial showed a striking 38% and 35% reduction in cardiovascular mortality and heart failure [HF] hospitalisation respectively. The exact mechanism (s) responsible for these effects remain (s) unclear. One potential mechanism is regression of Left ventricular hypertrophy (LVH).

**Methods:**

The DAPA-LVH trial is a prospective, double-blind, randomised, placebo-controlled ‘proof of concept’ single-centre study that has been ongoing since January 2017. It is designed specifically to assess whether the SGLT2 inhibitor dapagliflozin regresses left ventricular [LV] mass in patients with diabetes and left ventricular hypertrophy [LVH]. We are utilising cardiac and abdominal magnetic resonance imaging [MRI] and ambulatory blood pressure monitoring to quantify the cardiovascular and systemic effects of dapagliflozin 10 mg once daily against standard care over a 1 year observation period. The primary endpoint is to detect the changes in LV mass. The secondary outcomes are to assess the changes in, LV volumes, blood pressure, weight, visceral and subcutaneous fat.

**Discussion:**

This trial will be able to determine if SGLT2 inhibitor therapy reduces LV mass in patient with diabetes and LVH thereby strengthening their position as oral hypoglycaemic agents with cardioprotective benefits.

**Trial registration:**

Clinical Trials.gov: NCT02956811. Registered November 2016.

## Background

Patients with type 2 diabetes mellitus [T2DM] have double the risk of cardiovascular death [CVD] compared with patients without T2DM [1, 2]. Hyperglycaemia itself contributes to both the pathogenesis of atherosclerosis and heart failure [3]. While intensive glucose control reduces the risk of microvascular complications it appears to be insufficient to reduce cardiovascular [CV] events [4]. Three large randomized controlled trials [RCTs] ADVANCE, ACCORD and VADT failed to demonstrate any significant effect on macrovascular events of more intensive glycaemic control in patients with longstanding T2DM when compared with standard medical care [5–7].

The EMPA-REG OUTCOME trial was a landmark trial as it demonstrated for the first time that a glucose lowering agent could reduce CV events [8]. It was a multicentre, randomised, double blind, placebo-controlled trial performed in 7020 patients with T2DM at high cardiovascular risk comparing the SGLT2 inhibitor empagliflozin to placebo. In the empagliflozin group there were significantly lower rates of death from cardiovascular causes and heart failure hospitalisations, by 38 and 35% respectively. The exact mechanisms responsible for these effects remains unclear.

### Left ventricular hypertrophy

One potential mechanism is regression of left ventricular hypertrophy [LVH]. Left ventricular hypertrophy is thought to be present in up to 70% of patients with T2DM [9] It is a strong independent predictor of cardiovascular deaths and events and is even worse than triple vessel coronary disease [10, 11]. The reason why LVH is so adverse is because it predates so many different cardiovascular events i.e. LVH is intrinsically arrhythmogenic and causes sudden death, it impedes left ventricular [LV] filling and leads to diastolic heart failure, it reduces coronary perfusion reserve and causes ischaemia and it causes left atrial enlargement leading to atrial fibrillation [AF], and cardio-embolic strokes [12].

How does one cause regression of LVH? Controlling blood pressure [BP] and using a drug that blocks the renin-angiotensin system [RAS] are the standard approaches to the management of LVH but this approach is only partially effective since 44% of all patients with T2DM are normotensive patients with LVH [9]. Thus normotensive LVH is very common [9, 13]. Indeed, BP only contributes 25% to the variability in LV mass seen in a population [14]. Despite a “normal” BP, normotensive LVH is just as risky as is hypertensive LVH [15]. Nevertheless, we do know that regressing LVH irrespective of BP changes is an effective way to reduce the incidence of all major cardiovascular [CV] events including specifically sudden deaths, heart failure hospitalisations, new onset AF and strokes [16–23]. The LIFE trial provides conclusive proof that in diabetes, LVH regression per se reduces future cardiovascular events [by 24%], reduces CV deaths [by 37%] and reduces total deaths [by 41%] irrespective of BP [24].

Since controlling BP and using an angiotensin enzyme inhibitor or angiotensin receptor blocker is only partially effective at regressing LVH, we now need additional ways of regressing LVH. Insulin resistance is another mediator of LVH. The literature is awash with observational studies linking insulin resistance to LVH. The large studies are mostly positive which includes the Framingham Study, the Whitehall trial, the Strong Heart trial and the Women’s Health Initiative trial while HyperGEN is the one large negative trial [25–29]. Therefore, it is likely that glycaemia contributes to LVH. However, there is little evidence to date that glycaemic control alone affects the risk of CV events and thus key ancillary properties of each anti-glycaemic drug will be necessary to deliver the CV benefits we so badly need in diabetes [4, 30, 31]. A separate albeit related factor associated with LVH is also obesity [25, 32, 33].

### Sodium glucose linked co-transporter2 [SGLT2] inhibitors and their potential to regress LVH

In this study, we hypothesize that SGLT 2 inhibitors may be able to lead to LVH regression. Firstly, SGLT2 inhibitors employ a novel mechanism to lower blood glucose by enhancing urinary glucose excretion by competitively blocking the sodium glucose linked co-transporters in the proximal renal tubules, thus preventing the reabsorption of filtered glucose and sodium, resulting in glycosuria and natriuresis [34, 35]. This is in contrast with other antidiabetic medications which focus on restoring β-cell activity, insulin sensitivity and tissue glucose uptake to reduce plasma glucose levels. Accordingly, SGLT2 inhibitors are expected to maintain their potency as beta cell function declines with disease progression. Secondly, the glycosuric effects of SGLT2 inhibitors result in around 240-400 kcal/day loss through the urinary tract [36]. This calorific loss results in an average weight loss of around 2-3 kg that could help lead to LVH regression [37]. Finally, the natriuretic effect and subsequent osmotic diuresis could also be significant in patients with cardiovascular disease. This diuretic effect should reduce preload on the heart. The SGLT2 inhibitors lower blood pressure by 7-10 mmHg, reduce arterial stiffness and afterload [38–40].

In summary, SGLT2 inhibitors may improve cardiac structure because they appear to reduce the four main causes of LVH: glycaemia/insulin resistance, weight, preload and afterload [blood pressure] [41]. Our hypothesis is that dapagliflozin will regress LVH in normotensive patients with T2DM. If so, this could be a large part of explaining why such drugs reduce CV events in the EMPA-REG OUTCOME trial.

The issue of how SGLT2 Inhibitors reduces CV events in diabetes is a hot topic following EMPAREG OUTCOME. A large ongoing trial [DECLARE – TIMI 58] is also in progress assessing the effect of Dapagliflozin on CV events. If DECLARE-TIMI 58 shows clearly that dapagliflozin reduces CV events, then our trial if positive will have revealed a possible contributing mechanism to the reduced CV events i.e. LVH regression.

## Methods

### Study design

**Fig. 1:**
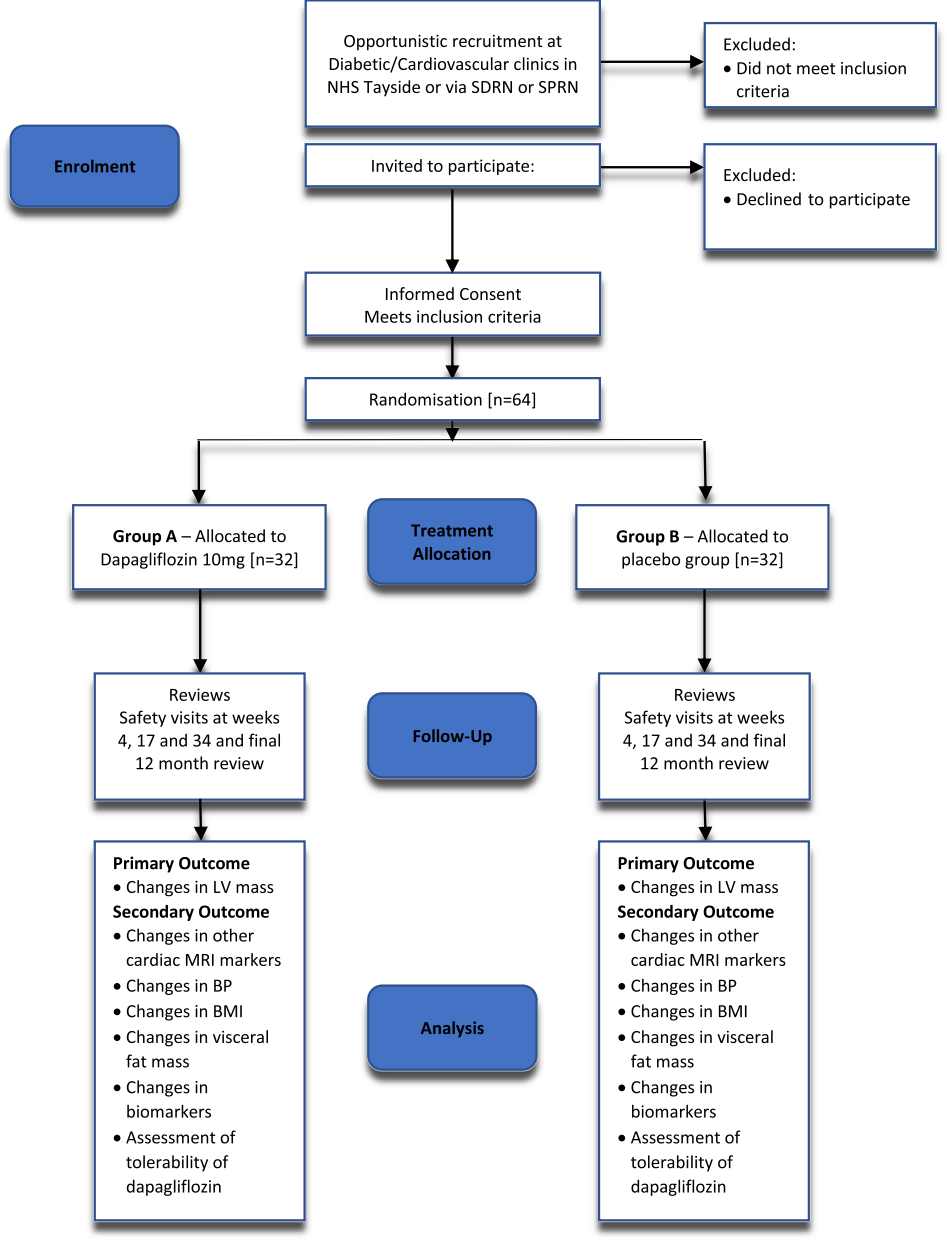
Study design flowchart. SDRN Scottish Diabetes Research Network; SPRN Scottish Primary Research Network; MRI Magnetic Resonance Imaging; BP Blood Pressure; BMI: Body Mass Index

The DAPA-LVH trial is a prospective, double-blind, randomised, placebo-controlled ‘proof of concept’ single-centre study conducted in NHS Tayside, Scotland designed to evaluate the efficacy of 12 months of the SGLT2 inhibitor dapagliflozin compared to placebo on left ventricular hypertrophy [LVH] in 64 normotensive participants with diabetes identified to have LVH. A recruitment window of 1.5 years from December 2016 has been set. Participants will be enrolled in this trial for a period of 12–13 months, [Fig. 1]. Therefore, the overall trial end date will be May 2019.

At the screening visit an initial medical history and clinical examination will be performed following informed consent. Participants will have an electrocardiogram performed and bloods taken for safety analysis. Vital signs including blood pressure will be recorded to confirm eligibility prior to enrolment. Blood pressure will be taken using an Omron M10-IT blood pressure monitor and eligible patients will have an office blood pressure of 145/90 mmHg averaged over three readings. Patients who require optimisation of their blood pressure will do so but will have to be stable on their current antihypertensive medications for 3 months prior to enrolment. Patients with borderline office blood pressure will undergo ambulatory blood pressure measurement (AMBP) This will be performed using a Spacelab 90,217 ambulatory blood pressure monitor. Inclusion will be possible with a 24 h mean blood pressure < 140/85 mmHg. Participants will also be screened for echocardiographic evidence of left ventricular hypertrophy [LVH] by the standard American Society of Echocardiology [ASE] criteria. This will be performed using a Philips Epiq 7 machine by a fully trained operator. Eligible participants identified to have LVH on echocardiography will be recruited. The full inclusion criteria are as listed below.

Recruited patients will return for a cardiac magnetic resonance imaging [CMRI] at the Clinical Research Centre, Ninewells Hospital, Dundee, within 3 weeks of the planned baseline [randomisation] visit. At the randomisation visit participants will have vital signs, body mass index, waist circumference and waist to hip ratio recorded. Participants will also be asked to undergo 24 h ambulatory BP monitoring using a Spacelab 90,217 ambulatory monitor. Examinations with greater than 50% successful readings will be deemed an acceptable exam. Bloods for safety analysis and research purposes [BNP, FIRI and Uric acid] will also be taken. During the visit, participants will also be randomly be assigned to either dapagliflozin 10 mg or matching placebo. The first dose will be administered during this visit and participants will be educated on the symptoms of both hypoglycaemia and diabetic ketoacidosis and given written instructions of how to manage it if either event occurs.

To reduce the likelihood of hypoglycaemia in participants taking insulin, participants who are already on insulin at time of recruitment will have their total daily dose of insulin reduced by 10% on the day they are randomised. Further dose titration will be done by the study team or GP based on the participant’s symptoms, home and laboratory-based blood sugar levels. Down-titration of therapy will be done in a stepwise manner starting with insulin. Other anti-diabetic agents will only be down-titrated once insulin has been discontinued.

In order to make the two groups comparable, a target HbA1c of ≤ 53 mmol/mol will be set for all participants. New onset diabetic patients will not be included in this study as SGLT2 inhibitors are currently only licensed as second line therapy. We will therefore be comparing a dapagliflozin [mostly as a second drug after metformin] based group against a conventionally treated group but without a SLGT2 inhibitor.

This will ensure that any difference in LV mass between groups is because dapagliflozin and all its ancillary cardiac properties and not because the two groups differed in glycaemic control.

With regards to BP, the main criteria will be that the baseline office BP is < 145/90 mmHg. However, the investigator will have clinical discretion to change anti-hypertensive drugs during the trial for safety reasons, under two circumstances. Firstly, if the systolic BP rises to above 140 mmHg on 2 consecutive visits during the trial then the participant can be started on extra anti-hypertensive drugs to re-achieve a systolic BP of < 145 mmHg. Secondly, if the participant suffers from dizziness and/or their systolic BP has fallen either by ≥ 25 mmHg or to an absolute level of ≤ 110 mmHg, then the attending physician can reduce or stop one of their antihypertensive drugs. These criteria serve two functions: firstly, to copy normal clinical practice and secondly to maintain participant safety.

Participants will return for three visits throughout the year to have safety and research bloods taken and to have vital signs, BMI, waist to hip ratio and waist circumference recorded. They will also be assessed for adverse events and to alter diabetic/antihypertensive therapy [if applicable].

The biomarker samples will be centrifuged and decanted into an aliquot which will be stored at -80 °C. Uric acid will be analysed with an elisa method using SIGMA-ALDRICH assay, UK. BNP will be measured by a MULTI-ARRAY system. The kit is from MESO SCALE DISCOVERY, USA. FIRI will be analysed with an elisa method using an ALPCO assay UK.

At the end of the 1 year study period, participants will return for repeated assessment of vital signs, BMI, waist circumference and waist to hip ratio, ambulatory blood pressure, echocardiography and CMRI. These values will be compared with their baseline tests to determine if any significant change has occurred with each of the two arms of the study populations. [See Table 1 for an overview of all visits scheduled for the trial].

**Table 1:** Overview of all the study visits in the DAPALVH trial

Visit	1 Screening	2 Baseline^a^	3 follow-up^a^	4 Follow-up^a^	5 Follow-up^a^	6 Last visit^a^
Timeline - weeks	0 to − 4	0 Within 4 weeks of screening visit	4^b^ [+/− 1 week]	17 [+/− 4 weeks]	34 [+/− 4 weeks]	52 [+/− 4 weeks]
Informed Consent	X					
Medical History	X					
Demographics	X					
Concomitant Medications	X	X	X	X	X	X
Physical Examination	X					
Height & weight	X					
BP & P	X	X	X	X	X	X
Temperature	X					
ECG	X					
Echo^c^	X					X
Safety Bloods^d^	X	X	X	X	X	X
Inclusion/Exclusion	X					
Pregnancy Testing if applicable^e^	X	X	X	X	X	X
Research Blood Sample ^f^		X	X	X	X	X
Genetic blood sample		X				
24 h BP		X				X
Cardiac & abdominal MRI^g^		X				X
Waist & hip measurement		X	X	X	X	X
Adjustment of diabetes medication		X	X	X	X	
Adjustment of anti-hypertensive medication		X	X	X	X	
Record Adverse Events		X	X	X	X	X
Randomisation		X				
Dispense Trial Drugs		X^h^	X	X	X	
Return trial drugs			X	X	X	X

^a^Participants will be fasted for these visits

^b^At least 3 weeks after commencing study medication

^c^If a participant has had a clinical echo done within the previous 6 months the results from this will be used for assessing eligibility, otherwise an echo will be performed at the screening visit

^d^U&E, FBC, LFT, cholesterol, HDL-cholesterol

^e^See section 7.2

^f^HbA1c, FIRI, BNP, glucose, uric acid. A sample to be held for future research will be taken at the last visit

^g^The MRI scan may be performed ± 3 weeks from the baseline visit and may therefore require a separate visit

^h^If the participant as not yet had their MRI scan they will be asked not to start their study medication until they have had their scan. Their Visit 3 will be delayed until the patient has had at least 3 weeks of study medication

### Study population

Diabetic patients with LVH will be identified for this study and all potential participants who meet the following criteria will be eligible for the trial:

Inclusion criteria:

- Diagnosed with type 2 diabetes mellitus based on the current American Diabetes Association guidelines.
- Aged 18–80 years
- Body Mass Index ≥ 23 kg/m^2^
- HbA1c 48-85 mmol/mol [last known result within in the previous 6 months]
- BP < 145/90 mmHg. Office BP at screening visit will be used however if this is above the inclusion criteria then the 24 h recording at screening visit will be used to confirm that in the opinion of the PI the BP is adequately controlled.
- Echocardiographic LV hypertrophy (defined as either an LV mass index of >115 g/m2 for men and > 95 g/m2 for women indexed to body surface area or > 48 g/m^2.7^ or 44 g/m^2.7^ when indexed to height^2.7^)
- Women of childbearing potential* [WoCBP] must agree to take precautions to avoid pregnancy throughout the trial and for 4 weeks after intake of the last dose

Exclusion criteria:

- Any condition that in the opinion of the investigator may render the participant unable to complete the trial including non CV disease [e.g. active malignancy].
- Participants with type 1 diabetes mellitus
- Participants who have previously had an episode of diabetic ketoacidosis.
- Serum Potassium or Sodium results outwith the normal range
- Diagnosis of clinical heart failure
- History of human immunodeficiency virus
- LV systolic dysfunction [LVEF < 45%] [last known result within in the previous 6 months]
- eGFR < 60 ml/min/1.73m^2^ [last known result within in the previous month] assessed using an abbreviated Modification of Diet in Renal Disease (MDRD) equation and indexed to 1.73m^2^.
- Known liver function tests > 3 times upper limit of normal [based on last measures and documented laboratory measurement in the previous 6 months]
- Body weight > 150Kg [unable to fit into a MRI scanner]
- Contraindications to MRI [e.g. claustrophobia, metal implants, penetrative eye injury or exposure to metal fragments in eye requiring medical attention]
- Past or current treatment with any SGLT2 inhibitor
- Allergy to any SGLT2 inhibitor or lactose or galactose intolerance
- Current treatment with loop diuretic
- Currently receiving long term [> 30 consecutive days] treatment with an oral steroid
- Pregnant or breast feeding participants
- Involvement in the planning and/or conduct of the trial [applies to Astra Zeneca or representative staff and/or staff at the trial site].
- Participation in another interventional study [other than observational trials and registries] within 30 days before visit 1.
- Individuals considered at risk for poor protocol or medication compliance

### Randomisation and treatment allocation

After successful screening for eligibility and safety, participants will be randomised to either dapagliflozin 10 mg or matching placebo [identical tablet containing lactose] in a double blind fashion. The double blind medication [dapagliflozin or placebo] will be prepared and packaged by AstraZeneca and labelled by our onsite clinical trials pharmaceutical pharmacy. Randomisation will be carried out via our Tayside Randomisation System [TRuST], a Good Clinical Practice [GCP] compliant web-based system run by the Tayside Clinical Trials Unit [TCTU], to preserve allocation concealment. This will securely backup both the randomisation seed and the randomisation allocation and have it available in the onsite 24 h emergency unblinding facility.

Once randomised, the participant will continue to take the trial medication once daily for 1 year, if tolerated. Compliance will be checked and documented, by the dispensing pharmacy, using tablet counts at each visit. If non-compliant, they will be encouraged to become compliant. If study drug needs to be stopped due to intolerance or adverse events, they will remain in the study in order to do an “intention to treat” analysis.

### Study outcomes

#### Primary objective and outcome

The primary outcome is to determine if dapagliflozin reduces left ventricular [LV] mass in patients with type 2 diabetes and LV hypertrophy when compared to placebo.

Secondary outcomes

- To determine if dapagliflozin has any effect on LV diastolic function.
- To determine if as expected dapagliflozin reduces blood pressure [BP]
- To assess the effect of dapagliflozin on left ventricular diastolic function and global longitudinal strain
- To determine if as expected dapagliflozin reduces body weight
- To determine if dapagliflozin reduces visceral fat mass
- To determine if as expected dapagliflozin reduces HbA1_C_.
- To determine the effect of dapagliflozin on B-type natriuretic peptide [BNP], Uric acid and Fasting Insulin Resistance Index [FIRI]
- To assess the tolerability of dapagliflozin in this patient group

#### Sample size and power calculations

For the primary outcome of LV mass regression using cardiac MRI, we have powered this study for an absolute change in LV mass based on previous studies that we have conducted in our unit. In our recently published study of LVH regression using allopurinol in participants with ischaemic heart disease [41], we found that allopurinol significantly reduced LV mass by − 5·2 ± 5·8 g compared to placebo − 1·3 ± 4·5 g [*p* < 0·007]. In per-cent terms, this degree of LVH regression is the same as seen between the two arms of the echo sub-study of the LIFE study where CV events were also different between groups. For an 80% power at a 5% significance level [α = 0·05], to detect a similar change in LV mass, we will require 29 subjects per group. Both our previous studies have shown a 10% dropout rate. Therefore, accounting for this, we will require a total of 64 participants [32 per group]. The 10% dropout rate is standard for such studies and includes those who died and those who withdraw consent.

#### Cardiac MRI protocol

Baseline and repeat Cardiac magnetic resonance imaging [CMRI] examinations at baseline visit [+/− 3 weeks] and after the final 12 month [+/− 4 weeks] visit will be performed on a 3 T Magnetrom Trio scanner [Siemens, Erlangen, Germany] using body array and spine matrix radiofrequency coils.. Short axis images from the atrio-ventricular ring to the LV apex will be acquired using a 2D ECG-gated breath hold segmented SSFP cine sequence with retrospective gating. Quantitative measurement of LV mass, ejection fraction (EF), end-diastolic volume (EDV), end-systolic volume (ESV) and stroke volume (SV) will be derived by region of interest contours placed around endocardial and epicardial LV borders at end systole and end diastole.

Transmitral flow and the isovolumetric relaxation time will be assessed using through plane phase contrast images with electrocardiographic synchronisation.

At the same time of the CMRI visceral and subcutaneous abdominal fat mass will be assessed. For measurement of subcutaneous adipose tissue (SCAT) and visceral adipose tissue (VAT) two successive axial 3D DIXON volume interpolated breath hold examination sequences will be acquired. These sequences will cover an anatomical area from the diaphragm to the pelvic floor, with a slice thickness of 3 mm and up to 88 slices (dependent on patient size) collected within a single 3D block.

For image analysis the ‘fat only’ DIXON MR images will be segmented using Analyze (Mayo Clinic) software, and the SCAT and VAT compartments are defined using a signal intensity threshold method with manual correction where required. Epicardial fat structures and fat associated with the vertebrae will both be omitted from the final calculated volumes.

From a single MRI slice at the L2-L3 intervertebral level. This single observer will analyse all the scans. Analysis will be performed offline [Argus Software, Siemens] by a single blinded observer.

The reproducibility of all parameters using MRI will be derived by this observer. A test-retest intra-observer co-efficient of variation of 2.0% is usual in this department’s past MRI studies. Should the scanner become unavailable for a prolonged period of time during the study an alternative scanner will be used. MRI methods will be adapted as appropriate to ensure optimal study results can be obtained.

## Discussion

**Fig. 2:**
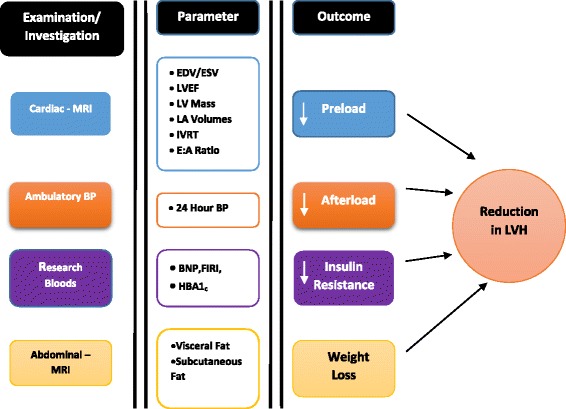
DAPA LVH trial hypothesis. The above figure explains the hypothesis of the DAPALVH trial where reduction in preload and afterload, weight and insulin resistance will all contribute to regression of left ventricular hypertrophy. This will be measured by cardiac MRI. BNP: B Type Natriuretic Peptide; BP: Blood Pressure; EDV: End Diastolic Volume; FIRI: Fasting Insulin Resistance Index; HBA1_C_: Glycosylated Haemoglobin; IVRT: Isovolumetric Relaxation Time; LA: Left Atrial; LV: Left Ventricular; LVEF: Left Ventricular Ejection Fraction; LVH: Left Ventricular Hypertrophy; MRI: Magnetic Resonance Imaging

SGLT2 inhibitors including dapagliflozin improve systemic glucose metabolism, lower blood pressure and lower body weight, thus they ameliorate the metabolic and haemodynamic risk factors heavily implicated in causing LVH. In this study, we propose that the SGLT2 inhibitors may be particularly suitable at regressing LVH. This effect might ultimately explain the reduced CV events seen so far in one large outcome trial with these drugs [Fig. 2].

The primary haemodynamic effect of SGLT2 inhibitors is an osmotic diuresis. Patients treated with dapagliflozin produce approximately 375mls of extra urine per day [36]. Several trials have shown that SGLT2 inhibitors lead to a reduction in systolic BP in a range of 3-5 mmHg and ~ 2-3 mmHg in diastolic BP [37]. This will be further assessed in our trial with ambulatory blood pressure recordings at randomisation and upon completion of the trial. The reason for the observed BP reduction with SGLT2 inhibition is not completely understood but is likely secondary to several different things including the modest diuretic effect, mild natriuresis and weight reduction [39, 42]. Data from a mechanistic trial has also demonstrated that empagliflozin reduced arterial stiffness in patients with type 1 diabetes mellitus [43]. These effects on intravascular volume and blood pressure will result in reduced preload and afterload respectively, thereby facilitating a reduction in intra-cardiac pressure and thereby an improvement in cardiac structure [24, 44]. Indeed following EMPA-REG Outcome trial there has been a lot of interest in the effects of SGLT2 inhibition on cardiac structure with a number of ongoing trials looking into the effects in patients with both diastolic and systolic heart failure in addition to cardiovascular outcomes [Tables 2, 3, and 4].

**Table 2:** Ongoing trials assessing the use of SGLT2 inhibitors in patients with systolic heart failure

SGLT2 Inhibitor	Trial Name; Clinical Trial Identifier	Primary Outcome measure	Patient Population^a^	Final Results
Empagliflozin	Empagliflozin Impact on Haemodynamics in Patients with Diabetes and Heart Failure [EMBRACE-HF]. [54] NCT03030222	Change in pulmonary artery diastolic pressure	*N* = 60 10 mg vs placebo Either LVEF 40% or > 40% NHYA II-IV HbA1_c_ ≥ 6.5% and ≤ 11% GFR > 30 ml/min	June 2018
Empagliflozin	SGLT2 Inhibition in Diabetic Patients With Heart Failure with Reduced Ejection Fraction [55] NCT02862067	SGLT2 inhibition effects on cardiorespiratory fitness	*N* = 31 10 mg/25 mg standard care LVEF ≤ 50% [in maximum tolerated HF therapy HbA1_c_ 7–10% Age ≥ 18 years GFR > 45 ml/min	June 2018
Empagliflozin	EMPagliflozin outcomE tRial in Patients with chrOnic heaRt Failure with Reduced Ejection Fraction [EMPEROR-Reduced] [56] NCT03057977	Time to first event of adjudicated CV death or adjudicated hospitalisation for HF in patients with HF with reduced ejection fraction	*N* = 2850 LVEF ≥ 36to ≤ 40%: NTproBNP ≥ 2500 pg/ml LVEF ≥ 31% to ≤ 35%: NT-proBNP ≥ 1000 pg/ml If LVEF ≤ 30% NT-proBNP ≥ 600 pg/ml Age > 18 years GFR > 20 ml/min	June 2020
Dapagliflozin	Dapagliflozin Effect on Symptoms and Biomarkers in Diabetes Patients with Heart Failure [DEFINE-HF] [57] NCT02653482	Differences in the average reduction of NTproBNP Proportion of patient that achieve a ≥ 5pts increase in heart failure disease specific quality of life score or a ≥ 20% decrease in NTproBNP	*N* = 250 10 mg vs placebo LVEF ≤ 40/NHYA II-III HbA1_c_ 6.5–11.0% Age 19–119 years GFR > 45 ml/min BNP ≥ 125 pg/ml and/or NTproBNP ≥ 600 pg/ml	May 2017
Dapagliflozin	Study to Evaluate the Effect of dapagliflozin on the Incidence of Worsening Heart Failure or Cardiovascular Death in Patients With Chronic Heart Failure With Reduced Ejection Fraction [Dapa-HF]. [58] NCT03036124	Time to first occurrence of the composite: CV death or hospitalisation for HF or urgent HF visit.	*N* = 4500 5/10 mg vs placebo LVEF ≤ 40/NHYA II-IV Age 18 to 130 years GFR > 30 ml/min NTproBNP ≥ 600 pg/ml	December 2019
Dapagliflozin	Safety and Effectiveness of SGLT2 inhibitors in Patients with Heart Failure and Diabetes [REFORM] [59] NCT02397421	Change in LV end systolic volume or LV end diastolic volume as determined by CMRI	*N* = 56 10 mg vs placebo. LVEF < 50%/NYHA I-II HbA1_c_ > 6% Age 18 to 75 years GFR > 45 ml/min	August 2017
Canagliflozin	A Randomised Active-Control Double-Blinded Study to Evaluate the Treatment of Diabetes in Patients with Systolic Heart Failure. [60] NCT02920918	Change from baseline aerobic exercise capacity Change from baseline ventilator efficiency	*N* = 88 LVEF ≤ 40%/NYHAII-III HbA1_C_ 6.5%–10% Age ≥ 18 years GFR >50 ml/min	November 2018

^a^Enrolment details correct at the time of writing as per ClinicalTrials.gov

*BNP* B Type Natriuretic Peptide, *CMRI* Cardiac Magnetic Resonance Imaging, *ESKD* End Stage Kidney Disease, *GFR* Glomerular Filtration Rate, *HbA1*_*C*_ Glycosylated Haemoglobin, *HF* Heart Failure, *LV* Left Ventricular, *LVEF* Left Ventricular Ejection Fraction, *NTproBNP* N Terminal pro brain natriuretic peptide, *NYHA* New York Heart Association, *SGLT2* Sodium Glucose Linked Co-Transporter2

**Table 3:** Ongoing Trials assessing the use of SGLT2 in patients with left ventricular hypertrophy or heart failure with preserved ejection fraction

SGLT 2 Inhibitor	Trial Name; Clinical Trial Identifier	Primary Outcome Measure	Patient Population^a^	Final Results
Empagliflozin	Effects of Empagliflozin on Left Diastolic Function Compared to Usual Care in Type 2 Diabetics [EmDia]. [61] NCT02932436	Difference in E/E’ ratio measured by echocardiography	*N* = 264 10 mg vs placebo Age 18–84 years HbA1_C_ ≥ 7–10% on diabetic therapy or ≥ 7–9% diet controlled GFR > 60 ml/min	October 2017
Empagliflozin	SGLT2 Inhibition and Left Ventricular Mass [EMPATROPHY] [62]; NCT 02728453	Change in ventricular mass assessed using CMRI	*N* = 60 25 mg vs 2-4 mg Glimepiride LVEF ≥ 45% Age ≥ 40 and < 80 years Office BP ≤ 150/95 mmHg HbA1_C_ 6.5–9% GFR > 60 ml/min	April 2018
Empagliflozin	Effects of Empagliflozin on Cardiac Structure in Patients with Type 2 Diabetes [EMPA-HEART] [63] NCT02998970	Left Ventricular Mass changes measured by CMRI at 24 weeks	*N* = 90 10 mg vs placebo LVEF > 30% Age ≥ 40 and ≤ 80 years HbA1_C_ 6.5- ≤ 10% GFR > 60 ml/min	June 2017
Dapagliflozin	Effects of Dapagliflozin on Biomarkers, Symptoms and Functional Status in Patients With Type 2 Diabetes or Pre-diabetes, and PRESERVED Ejection Fraction; [64] NCT030302235	Changes from baseline in NTproBNP	*N* = 320 10 mg vs placebo LVEF ≥ 45% Age 19 to < 119 years HbA1_C_ ≥ 5.7 - < 11% GFR < 30 ml/min	March 2019
Dapagliflozin	Does Dapagliflozin Regress Left Ventricular Hypertrophy in Patients with Type 2 Diabetes; [65] NCT02956911	Left Ventricular Mass changes measured by CMRI at 52 weeks	*N* = 64 10 mg vs placebo LVEF ≥ 45% Age ≥ 18 and ≤ 75 years HbA1_C_ ≥ 7 - < 10% GFR > 60 ml/min	May 2019

^a^Enrolment details correct at the time of writing as per ClinicalTrials.gov

*CMRI* Cardiac Magnetic Resonance Imaging, *GFR* Glomerular Filtration Rate, *HbA1*_*C*_ Glycosylated Haemoglobin, *HF* Heart Failure, *LV* Left Ventricular, *LVEF* Left Ventricular Ejection Fraction, *NTproBNP* N Terminal pro brain natriuretic peptide, *NYHA* New York Heart Association, *SGLT2* Sodium Glucose Linked Co-Transporter2

**Table 4:** Ongoing Cardiovascular Outcome Trials with SGLT2 inhibitors

SGLT2 Inhibitor	Trial Name; Clinical Trial Identifier	Primary Outcome Measure	Patient Population^a^	Final Results
Dapagliflozin	Dapagliflozin Effect on Cardiovascular Events. [DECLARE TIMI 58]; [66] NCT 01730534	CV Death, non-fatal MI, non-fatal ischaemic stroke	*N* = 17,276 10 mg vs placebo HbA1_C_ range not specified Age ≥ 40 years High CV risk	2019 [Estimated]
Canagliflozin	Canagliflozin Cardiovascular Assessment Study. CANVAS]; [67] NCT 01032629	CV Death, non-fatal MI, non-fatal ishaemic stroke	*N* = 4422 100 mg/300 mg vs placebo HbA1_C_ 7–10.5% Age ≥ 30 years High CV risk	June 2017
Canagliflozin	Evaluation of the Effects of Canagliflozin on Renal and Cardiovascular Outcomes in Participants with Diabetic Nephropathy [CREDENCE] [68]; NCT 02065791	Time to first occurrence of an event in the primary composite of endpoint of ESKD, doubling of serum creatinine, renal or CV death.	*N* = 4200 100 mg vs placebo HbA1_c_ 6.5–12% Age > 30 years GFR ≥ 30 to < 90 ml/min	June 2019

^a^Enrolment details correct at the time of writing as per ClinicalTrials.gov

*ESKD* End Stage Kidney Disease, *GFR* Glomerular Filtration Rate, *HbA1*_*C*_ Glycosylated Haemoglobin, *HF* Heart Failure, *LV* Left Ventricular, *LVEF* Left Ventricular Ejection Fraction, *NYHA* New York Heart Association, *SGLT2* Sodium Glucose Linked Co-Transporter2

Dapagliflozin is known to produce clinically meaningful reductions in HbA1_c._ [45] Studies have also shown that treatment with SGLT2 inhibitors improves insulin sensitivity as measured by peripheral glucose uptake [46, 47]. One such study showed that insulin mediated tissue glucose disposal increased by around 18% with only 2 weeks of dapagliflozin therapy [47]. Insulin resistance and hyperinsulinaemia have been associated with increased atherosclerosis risk and left ventricular hypertrophy [25–29, 48].

Other metabolic effects of the SGLT2 inhibitors include weight loss. With selective SGLT2 inhibition urinary glucose is increased resulting in a negative energy balance and subsequent weight loss [36]. A 24 week study comparing dapagliflozin to placebo showed a 2.5–3.5 kg weight reduction as a result of the calorific loss produced by glycosuria [49]. This is a finding throughout the SGLT2 class [37]. Of potential greater interest is how they change visceral fat mass as this is associated with an increased risk of T2DM and increased risk of CVD and overall mortality [50]. Indeed all the three currently available SGLT2 inhibitors when compared to glimepiride in dedicated body composition studies have shown that the majority of weight loss associated with SGLT2 inhibition was due to a reduction in visceral fat or subcutaneous fat [45, 51, 52]. Accordingly, we have chosen to also measure visceral and subcutaneous fat mass as a secondary outcome of the DAPA-LVH.

Given these metabolic and haemodynamic effects our hypothesis is that we will see a reduction in left ventricular mass. Indeed, pre-clinical work has shown that SGLT2 inhibitors are capable of reducing LV mass in a rat model with progressive HF [53]. We have therefore selected CMRI measurements of LV mass as our primary outcome measures for the DAPALVH Trial. By ensuring the trial is adequately powered we will determine if treatment with an SGLT2 inhibitor is able to reduce LV mass in diabetic patients with LVH.

The EMPA-REG Outcomes trial revealed a reduction in cardiovascular death and HF hospitalisations with the use of empagliflozin in patients with T2DM. However, it is unknown if these effects are seen throughout the SGLT2 inhibitor class. Other cardiovascular outcome trials such as DECLARE-TIMI 58 for dapagliflozin and CANVAS for canagliflozin will reveal whether the cardioprotective effects of SGLT2- inhibitor therapy is seen across the drug class. As described above this study will provide insights into the mechanism of the positive cardiovascular effects conferred by SGLT2 inhibitor therapy and may also help decide the course of future research – should LVH be a favoured target?

### Limitations

Firstly, this is a relatively small, single centre trial. The use of CMRI though has allowed the power of the trial to be preserved despite the small number of participants. However, given the small numbers some differences observed may still be the result of chance. Secondly, diabetes is a dynamic disease as a patient’s glycaemia control may fluctuate and this may necessitate dose adjustments of anti-diabetic medications during the trial which may confound the outcome. However, every measure will be taken to ensure blinding of the investigators is maintained and uniformity in the dose adjustments made.

## Conclusion

Historically much attention has focused on the prevention and treatment of the microvascular complications of diabetes. CVD however is still the main co-morbid condition and primary contributor to mortality in patients with diabetes. Besides metformin therapeutic options to optimise glycaemic control which reduce cardiovascular risk are limited. Empagliflozin an SGLT2 inhibitor has been shown to produce significant reductions in cardiovascular mortality and hospitalisation with heart failure [8]. We propose that SGLT2 inhibitors may cause regression of LVH due to their ability to reduce preload/afterload, weight and insulin resistance which may account for their positive cardiovascular effects. Upcoming major trials will establish if the effect seen with empagliflozin is an SGLT2 class effect. If so, the results of this study if positive will help us understand the mechanisms of the cardioprotective effects of SGLT2 inhibitors and if positive further establish this group of medications as anti-diabetic agents with the added value of protecting the heart.

## Abbreviations

ABMP: Ambulatory blood pressure measurement
AF: Atrial fibrillation
ASE: American society for echocardiography
BMI: Body mass index
BNP: B type natriuretic peptide
BP: Blood pressure
CMRI: Cardiac magnetic resonance
CVD: cardiovascular death
EDV: End diastolic volume
eGFR: Estimated glomerular filtration rate
ESV: End systolic volume
FBC: Full blood count
FIRI: Fasting insulin resistance index
GCP: Good clinical practice
HbA1C: Glycosylated haemoglobin
HDL: High density lipoprotein
LFT: Liver function tests
LV: Left ventricular
LVEF: Left ventricular ejection fraction
LVH: Left ventricular hypertrophy
MDRD: Modification of diet in renal disease
MRI: Magnetic resonance imaging
NTproBNP: N-terminal pro brain natriuretic peptide
RAS: Renin angiotensin system
SCAT: Scottish diabetes research network
SDRN: Subcutaneous adipose tissue
SGLT2: Sodium glucose co-transporter 2
SPCRN: Scottish primary care research network
SV: Stroke volume
T2DM: type2 diabetes mellitus
TCTU: Tayside clinical trials unit
TRuST: Tayside randomisation system
U&Es: Urea and electrolytes
VAT: Visceral adipose tissue
WoCBP: Woman of child bearing potential
